# Plant growth in *Arabidopsis* is assisted by compost soil-derived microbial communities

**DOI:** 10.3389/fpls.2013.00235

**Published:** 2013-07-04

**Authors:** Lilia C. Carvalhais, Frederico Muzzi, Chin-Hong Tan, Jin Hsien-Choo, Peer M. Schenk

**Affiliations:** Plant-Microbe Interactions Laboratory, School of Agriculture and Food Sciences, The University of QueenslandBrisbane, QLD, Australia

**Keywords:** *Arabidopsis*, gene expression, iron deficiency, plant growth promotion, plant-microbe interactions, soil microbial communities

## Abstract

Plants in natural and agricultural environments are continuously exposed to a plethora of diverse microorganisms resulting in microbial colonization of roots and the rhizosphere. This process is believed to be accompanied by an intricate network of ongoing simultaneous interactions. In this study, we examined *Arabidopsis thaliana* roots and shoots in the presence or absence of whole microbial communities extracted from compost soil. The results show a clear growth promoting effect on *Arabidopsis* shoots in the presence of soil microbes compared to plants grown in microbe-free soil under otherwise identical conditions. Element analyses showed that iron uptake was facilitated by these mixed microbial communities which also led to transcriptional downregulation of genes required for iron transport. In addition, soil microbial communities suppressed the expression of marker genes involved in nitrogen uptake, oxidative stress/redox signaling, and salicylic acid (SA)-mediated plant defense while upregulating jasmonate (JA) signaling, cell wall organization/biosynthesis and photosynthesis. Multi-species analyses such as simultaneous transcriptional profiling of plants and their interacting microorganisms (metatranscriptomics) coupled to metagenomics may further increase our understanding of the intricate networks underlying plant-microbe interactions.

## Introduction

Microbes and plants can establish a multitude of interactions with one another. From an agronomic perspective, soil microorganisms can exert beneficial or detrimental effects on plant growth and productivity. Many beneficial microbes were extensively studied. The classical examples are mycorrhizal fungi and rhizobia. Mycorrhizae associate with roots and provide phosphate, nitrogen (N) and water at the expense of photosynthates (Parniske, 2008; Feddermann et al., 2010; Garg and Chandel, 2010). Rhizobia fix N in exchange of carbon (C) sources especially in leguminous plant species (Raposeiras et al., 2006; Franche et al., 2009; Masson-Boivin et al., 2009). Other interactions involve root and rhizosphere-colonizing fungi and bacteria that are typically attracted by root exudates (Dennis et al., 2010; Carvalhais et al., 2011) and exert beneficial effects on plants by a number of mechanisms. These microbes are known as plant growth promoting microorganisms (PGPM) and they typically promote plant growth and/or improve health by a variety of mechanisms, including phosphate solubilization (Richardson et al., 2009), IAA production (Spaepen and Vanderleyden, 2011), siderophore biosynthesis (Dey et al., 2004), antibiotics production (Chen et al., 2009; Scholz et al., 2011), ACC deaminase activity (Glick et al., 2007; Siddikee et al., 2010), and can increase photosynthetic efficiency (Zhang et al., 2008) and induce systemic resistance (Wang et al., 2009; Phi et al., 2010; Zamioudis and Pieterse, 2012) in plants.

The effects of individual beneficial microbial isolates on plant growth and health have been widely reported (Scotti et al., 2007; Burkett-Cadena et al., 2008; Gulati et al., 2010; Hayat et al., 2010; Niu et al., 2011). The best documented bacterial genera of PGPM are *Pseudomonas* spp. (Jan et al., 2011; Yang et al., 2011) and *Bacillus* spp. (Idris et al., 2007; Fan et al., 2012; Perez-Garcia et al., 2011). Inoculation of mixtures of different strains has been also applied in attempts to produce synergistic results (Ryu et al., 2007; Zachow et al., 2010; Gupta et al., 2011). Plant gene expression during such interactions has also been evaluated in several instances. For example, transcriptome analyses of *Arabidopsis* plants colonized by the endophytic plant growth promoting rhizobacteria (PGPR) *Pseudomonas fluorescens* FPT9601-T5 (Wang et al., 2005), *Pseudomonas thivervalensis* (strain MLG45) (Cartieaux et al., 2003) as well as *Bradyrhizobium* sp. strain ORS278 and the pathogen *Pseudomonas syringae* pv. *tomato* DC3000 (Cartieaux et al., 2008) were performed. In another instance, gene expression profiles of cotton plants treated with the PGPR *Bacillus subtilis* UFLA285 were evaluated (Medeiros et al., 2011).

Pathogen/microbial associated molecular patterns (PAMPs or MAMPS) are invariant microbial epitopes that are recognized by plants. Cell surface elements including components of fungal cell wall (glucan, chitosan), lipopolysaccharides and peptidoglycanes, as well as flagellins are PAMPs/MAMPs that are recognized by receptors on the root cell surface and trigger a basal immune response, also known as PAMP-triggered immunity (PTI) (Jones and Dangl, 2006; Millet et al., 2010; Torres, 2010). It has been shown that early responses to infection by symbiotic organisms or pathogenic microbes are rather similar. Plants produce reactive oxygen species (ROS) in early stages of symbiosis with bacteria and fungi, and this is believed to be reminiscent of the oxidative burst generally triggered by pathogens (Torres, 2010). A defense response is initially produced, but then interrupted at a later stage (Zamioudis and Pieterse, 2012). Microbial molecules released extracellularly, such as siderophores (Meziane et al., 2005; Ran et al., 2005), antibiotics (Weller et al., 2002; Ongena et al., 2007), N-alkylated benzylamine (Ongena et al., 2005), N-acyl-l-homoserine lactone (Schuhegger et al., 2006), and volatiles (Ryu et al., 2004) have also been reported to elicit resistance. A body of evidence indicates that these systemic responses induced by beneficial rhizobacteria are typically mediated by JA as well as ethylene and can lead to induced systemic resistance (ISR) (Van Wees et al., 2008; Van der Ent et al., 2009; Niu et al., 2011). These induced systemic responses confer an enhanced defensive capacity on plants to subsequent pathogen infections and is also known as “priming.” JA also modulates responses against necrotrophic pathogens, which feed on dead tissues (Thomma et al., 2000) and JA limits the production of ROS in plants, contributing to resistance against necrotrophs (Ton et al., 2002; Pieterse et al., 2009). Alternatively, biotrophic pathogens that feed on living tissues induce salicylic acid (SA)-mediated responses when recognized, typically leading to a hypersensitive response, characterized by the production of ROS (Jones and Dangl, 2006; Pieterse et al., 2009). In most natural and agricultural environments, however, an intricate network of interactions between plants and their associated microbes takes place simultaneously and often successful pathogens hijack a defense pathway that worsens the infection or are falsely recognized as beneficial (Grant et al., 2013).

A number of studies have comprehensively characterized the root microbiome (Mendes et al., 2011; Bulgarelli et al., 2012; Lundberg et al., 2012). However, to our knowledge, there is currently no information available on transcriptional profiles of roots and shoots affected by whole microbial communities. In this study, we investigated transcriptional responses in roots and shoots of *Arabidopsis thaliana* ecotype Col-0 cultivated in sterile soil or soil inoculated with whole microbial communities extracted from compost soil. The results demonstrate that the combined effect of mixed microbial soil communities provides clear benefits to *Arabidopsis* nutrition and growth.

## Materials and methods

### Plant cultivation

Seeds of *A. thaliana* ecotype Col-0 were surface-sterilized using the vapour-phase sterilization method. Briefly, seeds were exposed to chlorine fumes in a desiccator jar for 4 h. Chlorine fumes were generated by adding concentrated hydrochloric acid to commercial bleach (minimum 10.5% available chlorine) up to a final concentration of 1%. Seeds were then placed onto half-strength Murashige and Skoog (MS) medium (pH 5.7) containing 0.8% agarose and incubated at 4°C in the dark for 72 h to break the dormancy. Plates with seeds were then transferred to a tissue culture room with a photoperiod of 16 h of light/8 h darkness and light intensity of 60–100 μmol m^2^ s^−1^ at 22°C for 14 days. Five seedlings each were then transplanted into 7.5 cm-diameter clear transparent tissue culture jars which contained 50 g of a 1:1 mixture of University of California mix and commercial compost soil (Greenfingers B2 potting mix, Nerang, Australia) that had undergone one of the soil treatments described below on the same day. This soil blend provided optimized water drainage for cultivation of *Arabidopsis* plants in tissue culture jars. Before planting, jars were filled with soil and γ-irradiated by using a ^60^Co source at a dose of 25 (KiloGray) kGy and a rate of 20 kGy/h. Sterile soils were tested for microbial contamination by incubating soil samples in Luria-Bertani and Potato Dextrose broth for 7 days at 30°C.

Treatments consisted of three biological replicates containing 10 jars each (50 plants per replicate). Four treatments were applied to sterile soil: (1) addition of non-sterile compost soil extract, which constituted a source of soil microbial communities, (2) filter-sterilized compost soil extract, (3) a sterile solution of Na-Fe-EDTA (13 μM) and (4) and sterile water. The compost soil extract was prepared by adding compost soil to sterile water up to a final concentration of 3.3% (w/v). After stirring, large particles were removed by filtering through Whatman grade 1 filter paper (11 μm). For the control, microbes were removed by passing this extract through a 0.22 μm filter. Each of the five seedlings in the tissue culture jars received 1.2 mL of the corresponding treatment solution. An additional experiment was carried out to compare autoclavation and γ-irradiation as soil sterilization methods. Soils were autoclaved twice at 121°C for 30 min and tested for microbial contamination as described above. This experiment also comprised three biological replicates per treatment with 50 plants per replicate (200 plants in total).

The use of compost soil extract as the inoculum allowed the addition of both culturable and unculturable soil microbes, as it is widely known that the vast majority of soil microbes cannot be cultivated in standard culture media (Kellenberger, 2001). A preliminary analysis on culturable bacteria from this compost soil showed that the majority came from the genera *Pseudomonas*, *Burkholderia*, *Pusillimonas*, and *Achromotobacter*. To also account for unculturable microbes, we performed 16 S rRNA gene amplicon pyrosequencing analysis using DNA extracted from a soil sample that has been inoculated with non-filtered compost soil extract (Carvalhais et al., unpublished results). This analysis targets both culturable and unculturable bacterial and archaea populations and a considerably high Operational Taxonomic Unit richness was found (~500).

### Plant harvest, total RNA extraction and cDNA synthesis

Four weeks after germination (including 2 weeks of soil treatments), plants were evaluated for phenotypic differences before harvesting. Soil was removed by washing and blotting on a tissue paper before fresh weights of roots and shoots were quickly measured, snap-frozen in liquid N, and stored at −80°C. Total RNA from roots and shoots was extracted independently with the SV Total RNA Isolation System (Promega, Madison, WI, USA) using 70 mg of ground tissue pooled from 50 plants per replicate as a representative sample. RNA concentrations were measured using a NanoDrop ND-1000 UV-Vis Spectrophotometer (Nanodrop Technologies, Rockland, DE). A total of 272 ng of RNA was used for cDNA synthesis using the SuperScript III reverse transcriptase for quantitative reverse transcriptase PCR (Invitrogen, Carlsbad, CA, US) following the manufacturer's instructions.

### Real-time quantitative reverse transcriptase PCR

Primers used in qRT-PCR were designed using the Primer Express software (Applied Biosystems, Foster City, CA, USA; Table S1). Each reaction was performed in a final volume of 10 μL, and contained 2 μL of cDNA, 1 μL of each primer (1 μM), 5 μL of SYBR Green using the 7900 HT Fast Real-time PCR system (Applied Biosystems, Foster City, CA, USA). Relative expression (*n*-fold) of the normalized target gene in both treatments was determined as proposed by Pfaffl (2001). *Arabidopsis* transcript levels in shoots and roots were normalized to the expression of a mixture of three genes encoding β *-ACTIN2*, *ACTIN7*, and *ACTIN8* (Schenk et al., 2005). Thermal cycling conditions consisted of 10 min at 95°C and 45 cycles of 15 s at 95°C and 1 min at 60°C prior to 2 min at 25°C.

### cDNA microarray analysis

Total RNA for microarray hybridizations was isolated from shoots and roots from an independent experiment with three biological replicates (50 plants each) as detailed above using γ-sterilized soil. Plant growth conditions were the same as the ones used for qRT-PCR. Three microarrays were used for the three replicate shoot samples and one microarray was used for a preliminary study on roots using combined RNA samples from three replicate root samples to obtain sufficient RNA. Total RNA was reverse-transcribed into cDNA and labeled with either Cy3 or Cy5 fluorescent dyes, mixed and used for subsequent hybridization onto 4 × 44K Agilent *Arabidopsis* GeneChip arrays (Agilent Technologies, Santa Clara, CA, USA). Labeling and hybridization of cDNA, including scanning of the chips were performed by the Australian Genome Research Facility (AGRF, Victoria, Australia). Signal intensities for each feature were extracted from scanned microarray images using Agilent Feature Extraction version 10.5.1.1 software (Agilent Technologies). Extracted data were analyzed using Integromics Biomarker Discovery (Integromics, Granada, Spain) and then normalized within arrays with the Loess algorithm and between arrays using the quantile method (Bolstad et al., 2003). Microarray data sets were deposited at Gene Expression Omnibus (accession GSE44984).

Significantly differentially expressed genes were selected based on the following criteria. Firstly, genes with signals that had high signal intensities, as well as higher than background signals based on the Agilent Feature Extraction in both Cy3 and Cy5 channels were selected. Secondly, genes with *P*-values lower than 0.05 using a parametric based test (Welch *t*-test) were considered statistically significant. Finally, genes that presented a signal difference of equal or greater than 1.5 fold-change in shoots and equal or greater than 2.5 fold-change in roots between the treatments (non-sterile vs. sterile) were considered as significant. A statistical analysis for overrepresentation of Gene Ontology (GO) terms in differentially expressed genes in shoots and roots in the presence of microbes was carried out for a simplified overview of affected functions. Gene IDs significantly associated with specific GO-terms (*P* < 0.05) were downloaded from the GO browser AmiGO (http://amigo.geneontology.org). Statistically significant microarray data was generally consistent with qRT-PCR data. However, differences were observed for three genes (*CAT3*, *AT2G43150* and *NIA1*). Out of these, *NIA1* was independently shown to be downregulated in the presence of microbes by qRT-PCR. Differences observed between microarray data and qRT-PCR may be caused by cross-hybridization, impurities in hybridization buffers causing deposition of dust on some spots and the fact that qRT-PCR and microarray data are derived from independent experiments where microbial communities may have slightly differed (although great care was taken to ensure that conditions were kept consistent).

### Plant tissue and rhizosphere soil element analysis

From each treatment, 28-day-old plants were harvested by careful uprooting and washing in water before blotting on tissue paper and drying at 70°C for 2 days. Three biological replicates containing 20 plants each were collected per treatment. Dried whole plant tissues were then ground to a fine powder and 200 mg of dry weight per replicate were used for subsequent analyses. Elemental analysis was carried out by inductively coupled plasma (ICP) atomic absorption with a Varian Vista Pro ICP Optical Emission Spectrometer (OES). Samples were digested in nitric/perchloric acid at a ratio of 5:1. The above procedure was repeated for corresponding rhizosphere soil that was collected by shaking the associated soil off carefully uprooted plants (Figure S1). Soil was then sieved through a 2 mm sieve to remove root residues (if any) before performing acid digestion. Total C and N concentration for plant tissues were separately determined by combustion using an automated dry combustion instrument LECO CNS 2000 (LECO, St. Joseph, MI, USA) at 1100°C. In addition, soil samples were analyzed using the DTPA (diethylenetriaminepentaacetic acid)-extraction method (Lindsay and Norvell, 1978) to determine bioavailable copper, iron, zinc, and manganese.

## Results

### Whole soil microbial communities promote arabidopsis shoot growth

**Figure 1:**
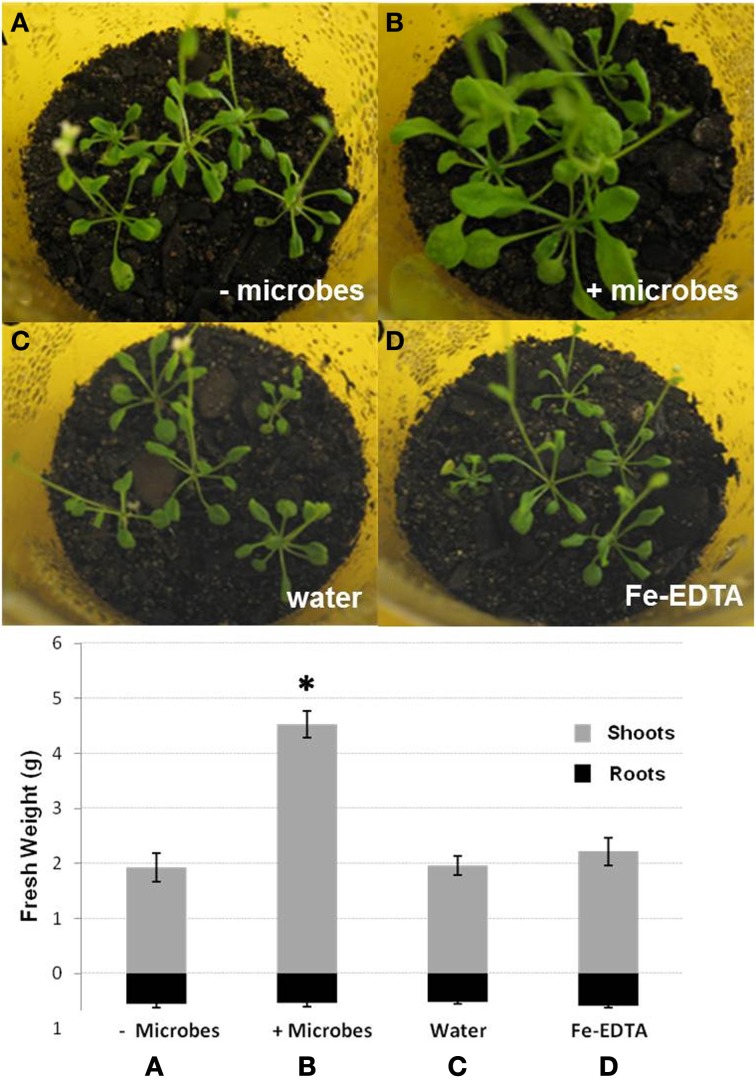
**Photographs of shoots and fresh weights of shoots and roots of *Arabidopsis* plants grown in sterile soil that was inoculated either with filter-sterilized soil extract (A; − microbes), non-sterile soil extract (B; + microbes), water (C) or Fe-EDTA (D)**. Bars represent mean values ±SE from three biological replicates (50 pooled plants/replicate). The asterisk indicates a statistically significant difference (*P* < 0.05).

To investigate the effects of whole soil microbial communities on *Arabidopsis* growth, plants were cultivated for 2 weeks in either sterile soil (also here referred to as “microbe-free soil”), or non-sterile soil (soil-containing microbes). For ideal comparisons, all soil was initially sterilized by γ-irradiation. These were then either inoculated with microbial extract from compost soil (non-sterile soil) or filter-sterilized extract from compost soil (microbe-free control). The addition of sterile soil extract was considered necessary to rule out any differences caused by the transfer of nutrients. In addition, the effect of the soil sterilization method used was evaluated by comparing plants grown in γ-irradiated as well as in autoclaved soils. Irrespective of the soil sterilization method used, plants cultivated in the presence of microbes displayed more vigorous growth than plants grown under sterile conditions. These plants had approximately twice the shoot weight (*P* < 0.05) and visibly larger leaf areas, while root biomass was not markedly different between treatments (*P* > 0.05; Figures 1A,B). Leaves of plants grown in sterile soil were also smaller, more poorly developed and fragile with signs of leaf curling, elongated leaf axes and crispy, brittle, but not dry, leaf structure (Figures 1, S1). Plants grown in sterile soil also often displayed a poorly developed caudal stem with developing inflorescences, while plants grown in the presence of microbes showed no signs of early flowering. Cotyledons of *Arabidopsis* plants in the absence of soil microbes were frequently yellow or dead (Figure S1), suggesting signs of early senescence in these plants. The above phenotypic differences were confirmed by independent additional experiments with three biological replicates (50 plants each) using autoclaved soil.

### Changes in nutritional status induced by microbes

**Figure 2:**
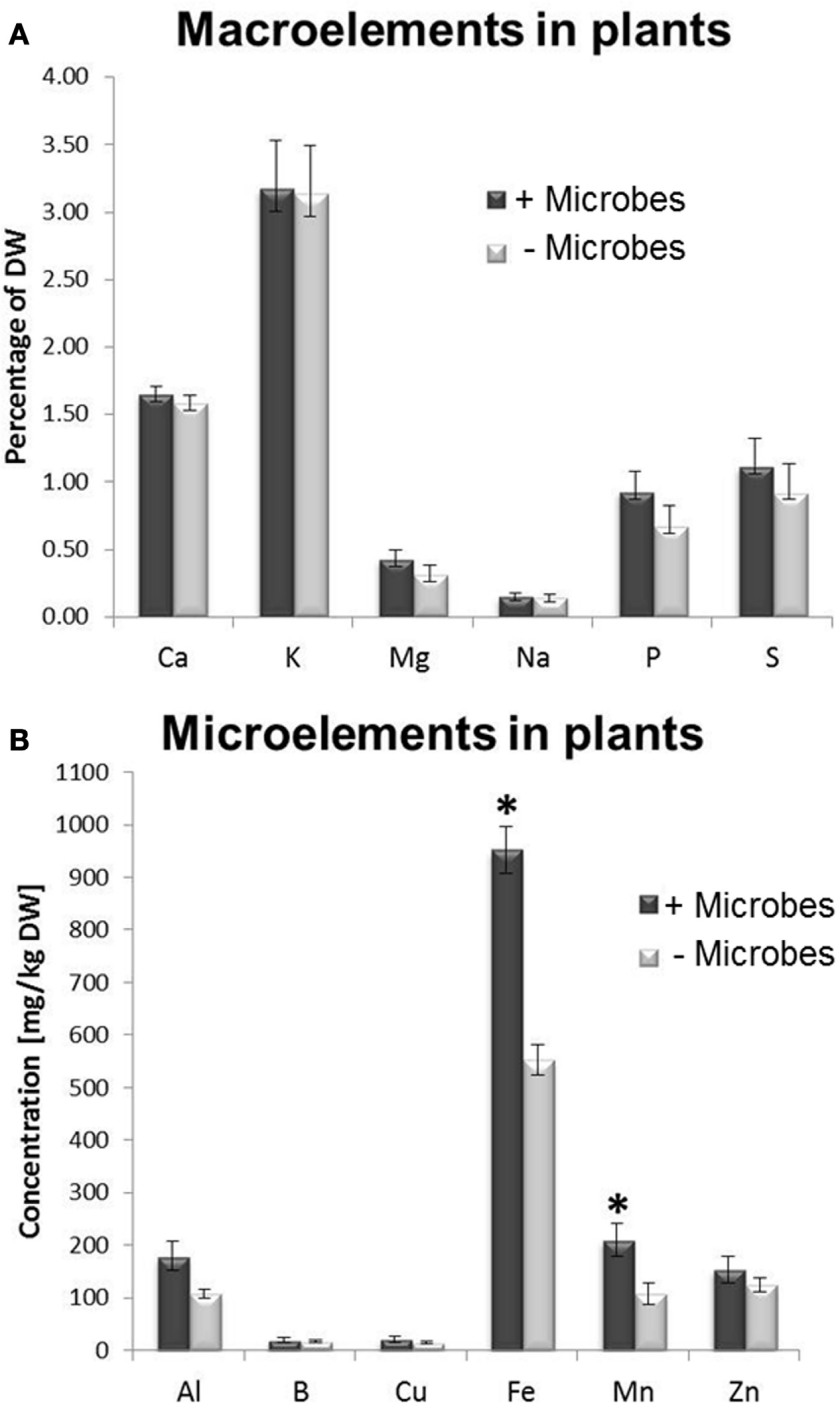
**Macro and micronutrient element analysis in *Arabidopsis* plants grown in the presence of soil microbes (non-sterile) or in microbe-free soils (sterile treatment). (A)** Macronutrients, **(B)** Micronutrients. Bars represent mean values per dry weight (DW) ± SE from three biological replicates (20 pooled plants/replicate). The asterisk indicates a statistically significant difference (*P* < 0.05).

A multi-element analysis of plant tissues was performed to investigate whether the enhanced growth in non-sterile soils resulted from a higher availability/acquisition of nutrients provided by microbes. No significant difference in carbon-nitrogen (C/N) ratio was observed in tissues from plants cultivated in sterile compared with non-sterile conditions (Figure S2). This implies that plant growth promotion was not associated with a higher N acquisition. However, a two-fold increase in Fe and Mn concentrations was found in plant tissues harvested from soils containing microbes compared to sterile soils (Figure 2). No significant differences in concentrations were found for other nutrients measured, such as calcium, potassium, magnesium, sodium, phosphate, sulphur, aluminium and boron (Figure 2).

**Figure 3:**
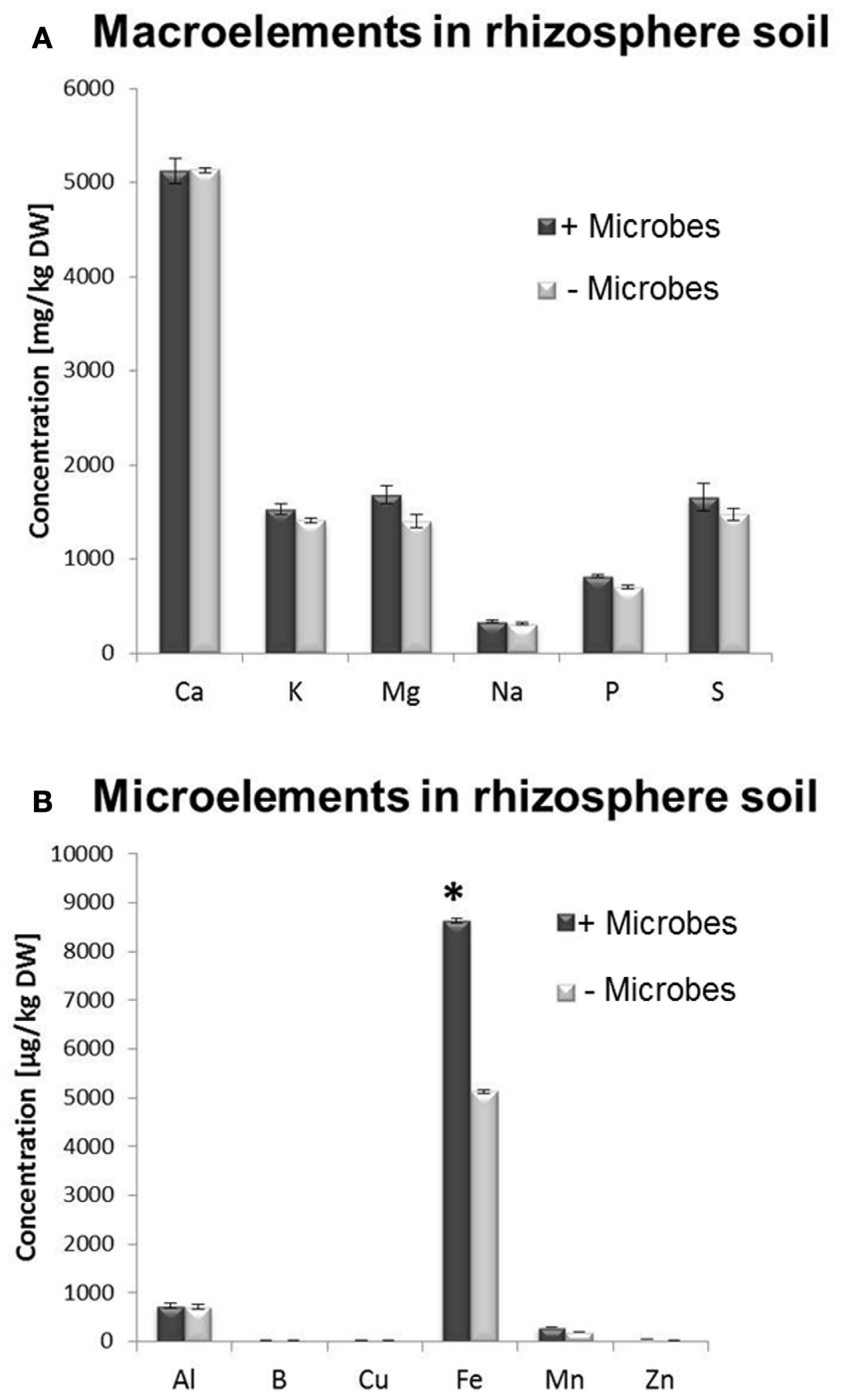
**ICP-OES analysis of total element concentration in sieved rhizosphere soil from *Arabidopsis* plants grown in the presence or absence of soil microbes. (A)** Macronutrients, **(B)** Micronutrients. Bars present mean values in mg/kg of soil dry weight (DW) ±SE from 3 independent replicates (100 g soil pooled from 10 pots/replicate). The asterisk indicates a statistically significant difference (*P* < 0.05).

**Figure 4:**
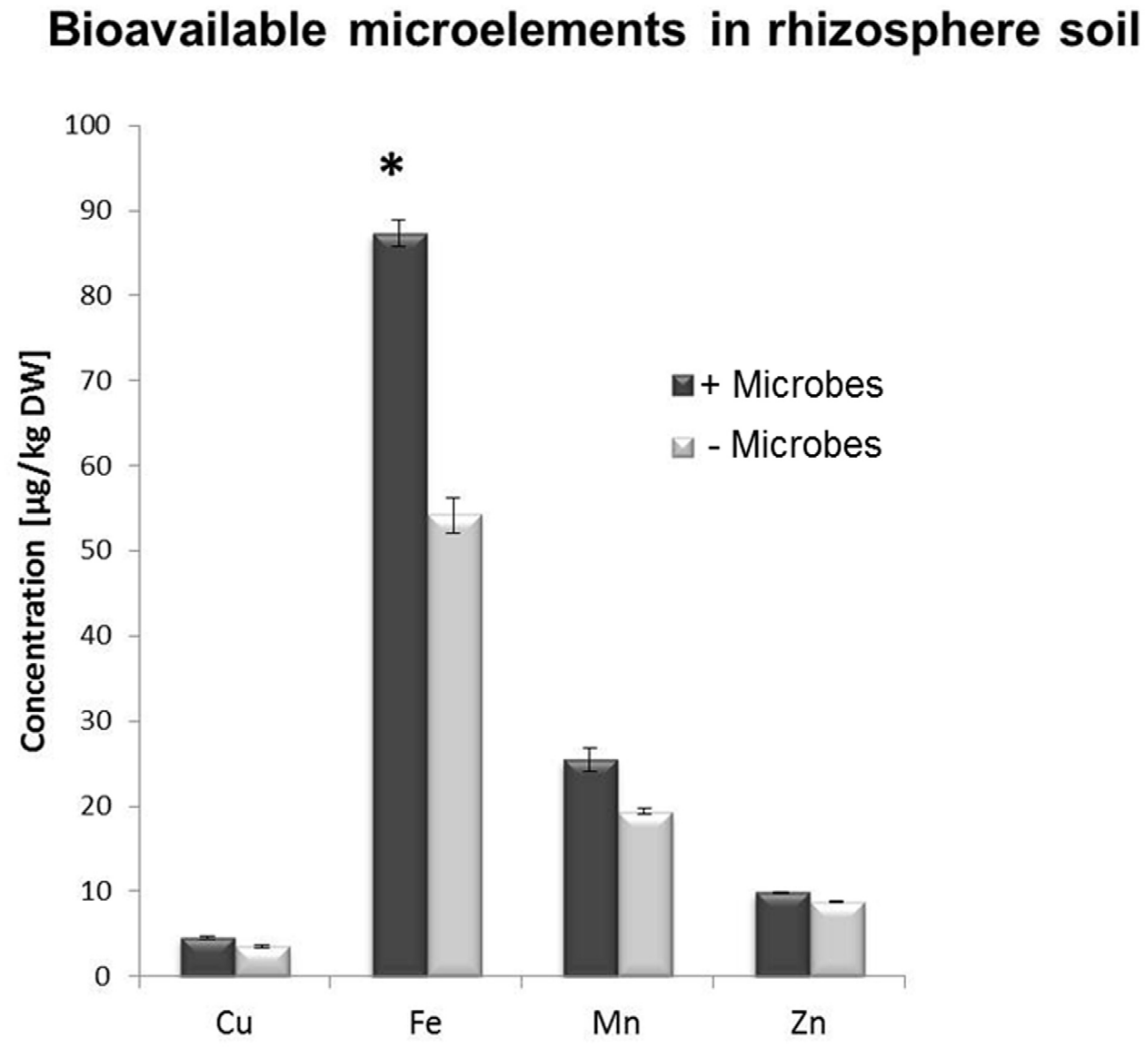
**DTPA extraction-based analysis of bioavailable trace element concentrations in rhizosphere soil from *Arabidopsis* plants grown in the presence or absence of soil microbes**. Bars present mean values in μg/kg of soil dry weight (DW) ±SE from 3 independent replicates (pooled rhizosphere soil from 50 plants/replicate). The asterisk indicates a statistically significant difference (*P* < 0.05).

A multi-element analysis (ICP-OES) was also carried out for bulk and rhizosphere soils to determine the nutrient status of the root-associated soil at the time of the harvest (28-day-old plants). Fe concentrations were significantly higher in non-sterile compared to sterile rhizosphere soils (Figure 3), while amounts of other elements were relatively similar and no significant differences could be observed for bulk soil distant from roots (Figure S3). A different method for micronutrient analysis (DTPA-extraction method) was also performed to measure bioavailable trace elements in rhizosphere soils. These confirmed that a higher concentration of Fe was available to plants in rhizosphere microbe-containing soils compared to microbe-free soils (Figure 4).

Fe-EDTA was added into sterile soils to investigate whether differences in *Arabidopsis* growth in non-sterile and microbe-free soils could be attributed solely to a higher availability of Fe. Water or filter-sterilized soil extract was used as controls. No significant differences in plant growth could be observed between additions of Fe-EDTA, sterile soil extract, or water (Figures 1C,D). These results indicate that a lower availability of Fe was not the only factor causing decreased shoot growth in microbe-free soils compared to non-sterile soils.

### Gene expression analysis of *Arabidopsis* plants grown in the presence or absence of soil microbes

Gene expression profiling was conducted on selected marker genes to identify processes involved in the interactions between *Arabidopsis* roots and soil microbial communities and to better understand the observed differences between plants grown in the presence or absence of soil microbes. The selection of genes for qRT-PCR was based on putative processes identified in the results of phenotypical analyses described previously, such as increased plant growth and enhanced iron incorporation. A number of marker genes associated to iron acquisition and metal homeostasis were chosen, such as *IRT1*, *FRO2*, *OPT3, MYB72*, and At3g07720. Although no differences in C/N ratio were observed in plant tissues (Figure S2), a gene involved in N acquisition (*NIA1*) was included as N is one of the major macronutrients required in plant nutrition. Given that beneficial microbes have been reported to alleviate stress derived from biotic and abiotic sources (de Zelicourt et al., 2013), genes that are representative of several stress-related responses were also selected, including pathogen defense responses (*PR1*, *PDF1.2, LECTIN1, LECTIN2, WRKY70, WRKY25, MYC2, ERF104*), oxidative stress responses (*CAT1*, *PER50, ERF6, ZAT10, OPR2*), abiotic stress (*WRKY25*, *MYB15*) and senescence (*SEN1*). A full list of selected marker genes, their locus names and qRT-PCR primers is shown in Table S1.

**Figure 5:**
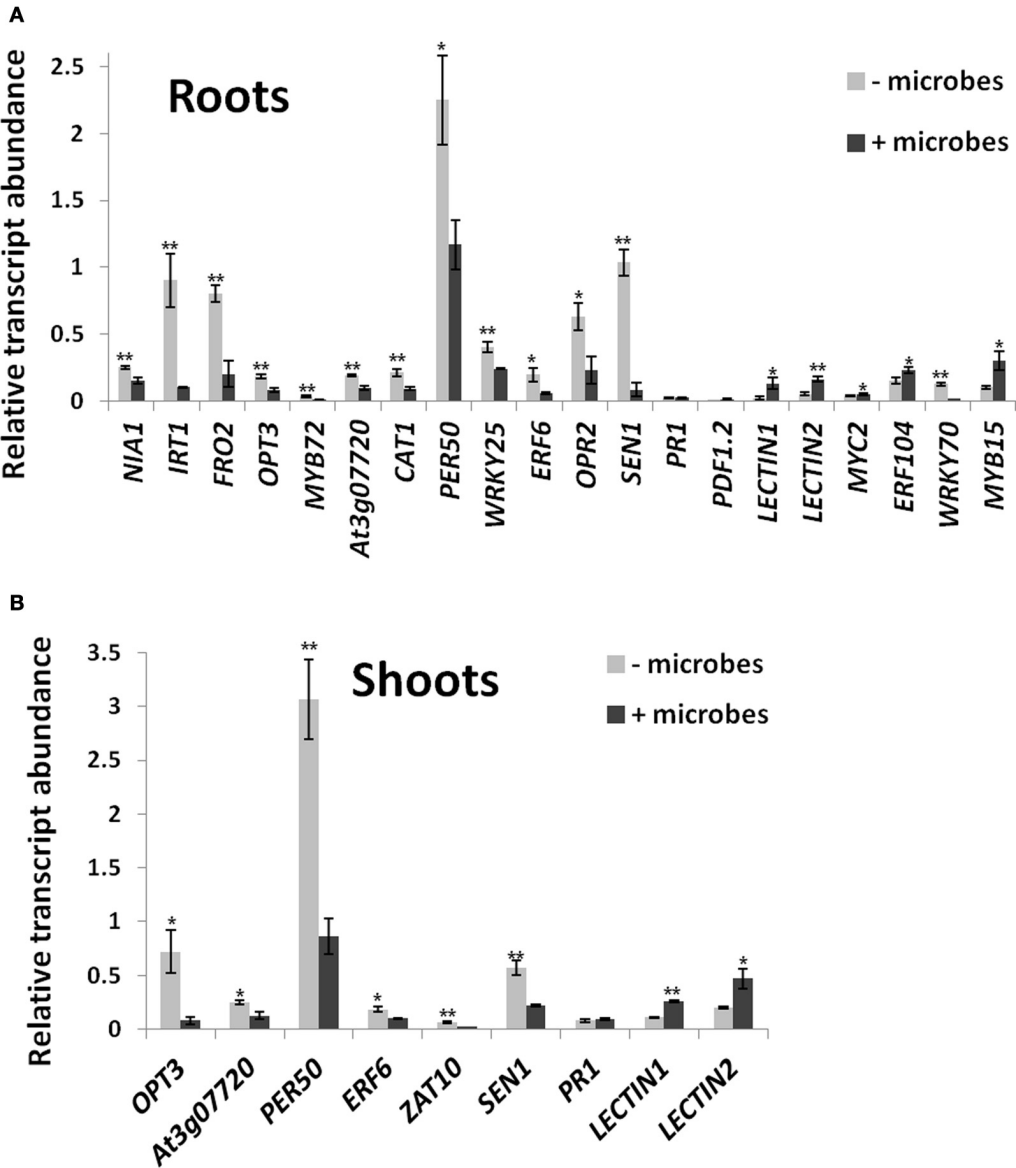
**Differential expression of marker genes in roots (A) and shoots (B) of *Arabidopsis* grown in soil in the presence or absence of whole microbial communities**. Transcript abundances are shown relative to *ACTIN* genes measured by qRT-PCR from three independent biological replicates. Each replicate contained pooled samples from 50 plants. Bars represent mean ± SE. The asterisk indicates a statistically significant difference (*P* < 0.05), two asterisks (*P* < 0.01). See Table S1 for full gene locus names.

*NIA1*, which is required for nitrate assimilation (Scheible et al., 2004), was downregulated in roots grown in the presence of microbes (Figure 5A). Genes directly involved in Fe acquisition (*IRT1*, *FRO2*) were also downregulated in roots of *Arabidopsis* roots grown in soil containing microbes (Figure 5A). Furthermore, genes involved in upstream signaling/regulation of Fe acquisition and metal homeostasis (*MYB72, OPT3*, *At3g07720*) were also downregulated (Figure 5A). qRT-PCR analysis of *IRT1* and *MYB72* using autoclaved instead of γ-irradiated soil gave similar results (Figure S4). This confirms that *Arabidopsis* plants were responding to the lower Fe availability in sterile soils compared to plants grown in the presence of microbes.

Genes involved in oxidative stress and redox homeostasis were generally lower expressed in roots when plants were grown in soil containing microbes. These include catalase (*CAT1*)- and peroxidase (*PER50*)-encoding genes that are required for ROS detoxification (Miller et al., 2008; Figure 5A). Furthermore, genes involved in upstream redox signaling (*WRKY25, ERF6; OPR2;* Zheng et al., 2007; Jiang and Deyholos, 2009; Wang et al., 2013) were also downregulated (Figure 5A). Similarly, *SEN1*, a gene involved in senescence, but also other pathways, including oxidative stress and SA-mediated plant defense (Schenk et al., 2005) was downregulated in roots in the presence of microbes (Figure 5A). Interestingly, the classical defense marker genes for the SA and JA defense pathways, *PR1* and *PDF1.2* (Thomma et al., 2000; Metraux, 2002) were not differentially expressed in roots, but their expression levels were also very low (<2% of *ACTIN* transcript levels; Figure 5A). JA signaling, a pathway also known to involve beneficial interactions with microbes for ISR (Van der Ent et al., 2009; Zamioudis and Pieterse, 2012), was upregulated in roots as shown by the induction of JA-regulated *LECTIN* genes (At3g15356 and At3g16530; Jung et al., 2007; Figure 5A) as well as the pathway's key regulatory gene *MYC2* (Anderson et al., 2004; Kazan and Manners, 2013), a gene that is also required for ISR triggered by beneficial soil microbes (Kazan and Manners, 2013). In addition, the JA/ethylene regulatory gene *ERF104* (Bethke et al., 2009) was upregulated, while *WRKY70*, a negative regulator of the JA pathway and a positive regulator of the SA pathway (Li et al., 2004), was downregulated in roots exposed to soil microbes (Figure 5A). The abiotic stress regulators *WRKY25* (SA-inducible; Zheng et al., 2007) and *MYB15* [abscisic acid (ABA)-inducible; Ding et al., 2009; Chinnusamy et al., 2010; Figure 5A] were also repressed.

Some of the physiological processes occurring in roots in the presence of soil microbes were also found in shoots. This includes downregulation of genes involved in Fe and metal homeostasis (*OPT3; At3g07720*), oxidative stress (*CAT1, PER50; ERF6; ZAT10*) and senescence (*SEN1*) Figure 5B). As in roots, *PR1* was not differentially expressed, while JA-responsive lectin-encoding genes At3g15356 and At3g16530 were induced in shoots in the presence of soil microbes (Figure 5B).

To identify other physiological processes associated with the presence of whole soil microbial communities that may lead to the increased shoot growth; a microarray analysis was carried on shoots and roots of *Arabidopsis* plants cultivated under the above conditions using γ-irradiated soil (Table S2). A GO enrichment analysis was performed in lists of up or downregulated genes to provide an overview of potential biological functions associated in soil microbe-root interactions (Tables 1, S3). In roots, GO terms that were enriched in the upregulated gene list, included response to stimulus, response to abiotic stimulus, response to oxidative stress, response to light stimulus, C fixation and plant-type cell wall organization or biogenesis (Table S3). In the downregulated gene list in roots, GO terms associated with iron transport and homeostasis were enriched (Table S3), which corroborates the results of the qRT-PCR (Figure 5A). In shoots, several GO terms were overrepresented in the upregulated gene list, such as those related to photosynthesis and responses to chemical and biotic stimulus, including response to other organisms as bacteria and fungi (chitin), stress related responses, JA signaling and ISR (Table 1). Most of these GO terms reveal responses involved in biotic interactions, which were apparently more pronounced in shoots compared to roots in terms of gene expression. Indeed, the overall phenotypical outcome of introducing a compost soil-derived community of microbes was an enhanced shoot growth, as opposed to no changes found in root length and biomass (Figure 1). Interestingly, several genes involved in defense responses were also up-regulated in shoots, as evidenced by the GO term enrichment analysis (Table 1). The enrichment of GO terms related to photosynthesis both in shoots and roots suggests that soil microbe-mediated plant growth promotion also coincided with increased photosynthetic activity. Most of the genes that contributed to the overrepresentation of the GO term “response to biotic stimulus” are also involved in defense responses, specifically jasmonate signaling (*CYP71A12*), SA signaling (*CRK4, AT5G02490, ATMPK3, WRKY70*), and oxidative stress (*At5G64120, RBOHD, At2G37130, ATMPK3*).

**Table 1:** **Identification of Gene Ontology (GO) functional categories that are enriched in transcript populations in shoots (*n* = 3) in the presence of whole soil microbial communities**.

**GO term**	***P*-value**	**Sample frequency**	**Background frequency**	**Gene names**
**UPREGULATED**				
GO:0050896 response to stimulus	1.06E-06	66/243 (27.2%)	3815/29970 (12.7%)	FHL, AT1G72920, VTC2, ACS6, AT5G64120, AT4G34810, LHCA1, CRK4, CYP71A12, RBOHD, WRKY38, AT5G38420, SUR1, WRKY46, CYP83B1, PRXR1, CYP83A1, AT2G04795, AT5G38410, PMSR2, ERF2, MYB51, AT4G09420, WRKY54, ATL2, AT3G26590, AT5G51470, AT1G78410, PIL1, CYP707A2, ATBBD1, ERF11, AT1G72910, AT1G02820, TCH2, AT1G20620, SHI, AT5G38344, APX1, JAZ6, NIP6;1, AT5G02490, AT3G02840, PUB22, AT1G74670, ERF104, SZF1, PUB23, ECS1, AT2G37130, NAXT1, AT1G70000, TIP2, AT5G51190, ERF4, MGD2, AT1G32920, STO, AT1G20823, AT4G24350, ATRLP26, EBF1, CYP71B2, ATMPK3, WRKY70, AT4G30370
GO:0009607 response to biotic stimulus	1.83E-02	18/243 (7.4%)	703/29970 (2.3%)	VTC2, AT5G64120, CRK4, CYP71A12, RBOHD, WRKY38, CYP83B1, ERF2, MYB51, ATBBD1, AT5G02490, AT3G02840, ERF104, AT2G37130, TIP2, ERF4, ATMPK3, WRKY70
GO:0042221 response to chemical stimulus	4.50E-03	36/243 (14.8%)	1984/29970 (6.6%)	VTC2, ACS6, AT4G34810, CRK4, WRKY38, WRKY46, PRXR1, AT2G04795, PMSR2, ERF2, MYB51, ATL2, AT3G26590, AT5G51470, AT1G78410, ERF11, TCH2, AT1G20620, SHI, APX1, JAZ6, NIP6;1, AT5G02490, AT3G02840, PUB22, AT1G74670, SZF1, PUB23, AT1G70000, AT5G51190, ERF4, AT1G20823, EBF1, ATMPK3, WRKY70,AT4G30370
GO:0009743 response to carbohydrate stimulus	1.72E-05	13/243 (5.3%)	209/29970 (0.7%)	WRKY46, ERF2, ATL2, PUB22, AT1G74670, SZF1, PUB23, AT5G51190, ERF4, AT1G20823, ATMPK3, WRKY70, AT4G30370
GO:0051707 response to other organism	7.87E-03	18/243 (7.4%)	660/29970 (2.2%)	VTC2, AT5G64120, CRK4, CYP71A12, RBOHD, WRKY38, CYP83B1, ERF2, MYB51, ATBBD1, AT5G02490, AT3G02840, ERF104, AT2G37130, TIP2, ERF4, ATMPK3, WRKY70
GO:0009617 response to bacterium	4.76E-03	12/243 (4.9%)	291/29970 (1.0%)	VTC2, CRK4, CYP71A12, WRKY38, CYP83B1, ERF2, MYB51, AT5G02490, TIP2, ERF4, ATMPK3, WRKY70
GO:0010200 response to chitin	5.37E-07	12/243 (4.9%)	127/29970 (0.4%)	WRKY46, ERF2, ATL2, PUB22, SZF1, PUB23, AT5G51190, ERF4, AT1G20823, ATMPK3, WRKY70, AT4G30370
GO:0006952 defense response	1.37E-05	25/243 (10.3%)	815/29970 (2.7%)	AT1G72920, VTC2, AT5G64120, CRK4, RBOHD, WRKY38, CYP83B1, ERF2, MYB51, AT4G09420, WRKY54, ATL2, ATBBD1, AT1G72910, TCH2, AT5G38344, PUB22, ERF104, PUB23, ECS1, AT2G37130, TIP2, ERF4, AtRLP26, WRKY70
GO:0006950 response to stress	1.02E-04	42/243 (17.3%)	2161/29970 (7.2%)	AT1G72920, VTC2, ACS6, AT5G64120, CRK4, RBOHD, WRKY38, CYP83B1, PRXR1, AT2G04795, PMSR2, ERF2, MYB51, AT4G09420, WRKY54, ATL2, AT1G78410, ATBBD1, AT1G72910, AT1G02820, TCH2, AT1G20620, AT5G38344, APX1, JAZ6, AT5G02490, PUB22, ERF104, PUB23, ECS1, AT2G37130, AT1G70000, TIP2, ERF4, MGD2, AT1G32920, STO, AT4G24350, AtRLP26, CYP71B2, ATMPK3, WRKY70
GO:0010033 response to organic substance	3.97E-03	26/243 (10.7%)	1181/29970 (3.9%)	VTC2, ACS6, AT4G34810, CRK4, WRKY38, WRKY46, ERF2, MYB51, ATL2, AT5G51470, ERF11, TCH2, SHI, JAZ6, PUB22, AT1G74670, SZF1, PUB23, AT1G70000, AT5G51190, ERF4, AT1G20823, EBF1, ATMPK3, WRKY70, AT4G30370
GO:0045730 respiratory burst	4.81E-03	3/243 (1.2%)	5/29970 (0.0%)	AT5G64120, PUB22, PUB23
GO:0015979 photosynthesis	8.47E-03	9/243 (3.7%)	166/29970 (0.6%)	LHCA1, LHCA2, LHB1B2, LHCA3, AT5G28450, LHCA4, PSAF, CAB1, PSI-P
GO:0042435 indole derivative biosynthetic process	1.62E-02	5/243 (2.1%)	40/29970 (0.1%)	SUR1, CYP83B1, MYB51, NIT1, ATMPK3
GO:0042430 indole and derivative metabolic process	3.25E-02	5/243 (2.1%)	46/29970 (0.2%)	SUR1, CYP83B1, MYB51, NIT1, ATMPK3
GO:0009753 response to jasmonic acid stimulus	4.76E-02	8/243 (3.3%)	160/29970 (0.5%)	VTC2, ACS6, ERF2, MYB51, JAZ6, AT1G70000, ERF4, WRKY70
GO:0009682 induced systemic resistance	4.96E-03	4/243 (1.6%)	15/29970 (0.1%)	CYP83B1, ERF2, ERF4, WRKY70
GO:0009864 induced systemic resistance, jasmonic acid mediated signaling pathway	1.66E-02	3/243 (1.2%)	7/29970 (0.0%)	ERF2, ERF4, WRKY70
GO:0006790 sulfur metabolic process	2.29E-02	9/243 (3.7%)	188/29970 (0.6%)	APR1, ACS6, SUR1, CYSD2, CYP83B1, APS3, CYP83A1, MYB51, ATMPK3
**DOWNREGULATED**				
GO:0050896 response to stimulus	1.20E-02	19/55 (34.5%)	3815/29970 (12.7%)	GT72B1, VTC2, RAP2.4, AT1G76190, CP12-2, HSF, A4A, AT1G70000, ATMRP7, CBL5, CRY1, AT2G31730, AT2G40460, STO, AT5G41750, AT3G23600, EBF1, PRXR1, RING1, YSL1
GO:0042221 response to chemical stimulus	4.36E-03	14/55 (25.5%)	1984/29970 (6.6%)	GT72B1, VTC2, RAP2.4, CP12-2, HSF, A4A, AT1G70000, CBL5, AT2G31730, EBF1, PRXR1, RING1, YSL1
GO:0009651 response to salt stress	3.33E-02	6/55 (10.9%)	386/29970 (1.3%)	GT72B1, RAP2.4, AT1G70000, CBL5, STO, AT3G23600

## Discussion

### Plant nutrition in the presence of rhizosphere microorganisms

The present study shows that plants grown in the presence of whole soil microbial communities exhibited enhanced shoot growth when compared to plants cultivated on sterile soil. This is consistent with related studies that reported that plants inoculated with individual beneficial microorganisms displayed an increase of fresh weight compared to axenically grown plants (Persello-Cartieaux et al., 2001; Ryu et al., 2005). However, so far it remained unclear whether this growth promoting effect can also be achieved by a complex assemblage of soil microbial populations. The increased shoot growth may be partially attributed to improved plant nutrition (Figures 2B, 3B, and 4). Global gene expression profiling using microarray analyses of shoots and roots also showed that many genes were down-regulated in the presence of soil microorganisms which may have translated into metabolic cost savings for these plants. Although the presence of soil microbes did not affect plant C/N ratio (Figure S2), the downregulation of *NIA1*, which is involved in nitrate assimilation (Scheible et al., 2004), indicates that microbes compete for the nitrate available in the soil, as reported previously (Song et al., 2007; Xu et al., 2011). Instead, plants may utilize other N sources available to them, including organic forms. Indeed, there are several ways that rhizosphere bacteria, many of which also fix atmospheric N_2_, contribute to N uptake in plants, including organic forms such as amino acids, oligopeptides, DNA, as well as whole proteins (Paungfoo-Lonhienne et al., 2008, 2010a). In addition, whole bacteria and yeast cells have been shown to be taken up and consumed by plant roots (Paungfoo-Lonhienne et al., 2010b), although it is currently uncertain how significant this process is to N acquisition.

Tissues as well as rhizosphere soils collected from plants grown in the presence of microbes showed higher Fe content (Figures 2, 3, 4). In addition, genes involved in Fe acquisition (e.g., the high affinity iron transporter IRT1; Colangelo and Guerinot, 2004) and metal homeostasis in these plants were down-regulated (Figure 5A). There are two strategies that plants utilize to acquire Fe in conditions of deprivation. Strategy I is employed by dicots and non-graminaceous monocots and it relies on reductases and proton secretion to increase the availability of insoluble inorganic iron by means of lowering the redox conditions and rhizosphere acidification. Conversely, Strategy II applies to graminaceous monocots that release chelating organic molecules known as siderophores to scavenge iron from the soil solution. Siderophores are also able to release iron from complexes contained in humic and fulvic acids present in the organic matter, as well as to mobilize Fe from minerals in the solid phase. Such molecules can be produced by nearly all cultured microbial isolates (Crowley, 2006) and especially microbe-derived siderophores have been reported to confer resistance to hydrogen peroxide (Dellagi et al., 1998; Oide et al., 2006). This may partly explain why plants in the absence of microbes displayed higher expression levels for genes involved in oxidative stress/redox homeostasis (*CAT1, PER50*; Figure 5A). In plants that adopt strategy I for iron acquisition; such as *Arabidopsis*, the plasma membrane-bound Fe^3+^ chelate reductase FRO2 catalyzes the reduction of Fe^3+^ at the cell surface (Robinson et al., 1999), which is then taken up by IRT1, an iron regulated transporter of the ZIP family (Varotto et al., 2002; Vert et al., 2002). Consistent with our findings, *IRT1, FRO2*, *MYB72* and *At3g07720* were previously found to be upregulated during Fe deficiency and are directly regulated by FIT, a basic helix–loop–helix (bHLH) transcription factor required for root iron uptake (Colangelo and Guerinot, 2004; Sivitz et al., 2012). MYB72 has a demonstrated role in iron uptake regulation (Sivitz et al., 2012), but interestingly is also activated by beneficial soil microbes (Van der Ent et al., 2008). *At3g07720* encodes a Kelch repeat protein and is regulated by Fe deficiency (Sivitz et al., 2012) but has putative biochemical functions including SA biosynthesis. OPT3 is also involved in metal ion homeostasis (Stacey et al., 2008). Upregulation of these genes in plants grown in the absence of microbes (Figure 5) is consistent with the finding that these plants did not have the same amount of bioavailable Fe than plants grown in the presence of microbes. This effect was observed irrespective of the soil sterilization method used (Figure S4). Although γ-irradiation is known for posing the least disturbance to physical and chemical properties in soils in comparison to autoclaving (Alef and Nannipleri, 1995; Berns et al., 2008), significant differential expression for *IRT1* and *MYB72* were maintained irrespective of the sterilization method used (Figure S4). Plant growth can be indirectly affected by siderophore-producing bacteria as they exhibit improved rhizosphere competence in Fe-deficient soils (Babalola, 2010). However, the addition of Fe-EDTA to the sterile soil did not trigger significantly increased plant growth compared to plants grown in microbe-free soil (Figures 1C,D). This indicates that iron supply was not the dominant factor associated to the enhanced plant growth in non-sterile conditions. Interestingly, Mn was also less abundant in *Arabidopsis* plants in the absence of microbes (Figures 3, 4). Indeed, both elements, Fe and Mn, use the same ITR1- and FRO2-mediated transport mechanisms for uptake in *Arabidopsis* plants (Colangelo and Guerinot, 2004; Sivitz et al., 2012). It is worth considering that *Arabidopsis* plants have been shown to incorporate Fe chelated to the microbial siderophore pyoverdine more efficiently than Fe chelated to EDTA (Vansuyt et al., 2007). Therefore, it appears possible that incorporation of Fe chelated to microbial siderophores may have contributed to the increased shoot growth of *Arabidopsis* as well as to the higher Fe concentration in plant tissues and rhizosphere soil.

### Oxidative stress, redox homeostasis and senescence

Beneficial plant–rhizobacteria interactions have been shown to alleviate plant abiotic stress conditions associated with oxidative stress (Dimkpa et al., 2009). Plants inoculated with known beneficial microbes generally show lower activities of antioxidant enzymes, such as catalases and peroxidases as compared to uninoculated plants (Bianco and Defez, 2009; Sandhya et al., 2010). In addition to these enzymes, several regulatory genes for oxidate stress signaling have been characterized in *Arabidopsis*, for example *ERF6*, *WRKY25*, and *ZAT10* (Wang et al., 2013; Zheng et al., 2007; Mittler et al., 2006). The lower expression of *CAT1, PER50, WRKY25, ERF6*, and *OPR2* in roots (Figure 5A) and of *CAT1, PER50*, *ERF6*, and *ZAT10* in shoots (Figure 5B) of plants grown in microbe-containing soil suggests that beneficial microbes may be present in the whole soil microbial communities used in this study. A marker gene involved in senescence (*SEN1*) that is highly expressed in cotyledons (Schenk et al., 2005) was also downregulated in leaves in the presence of microbes (Figure 5B). It should be noted that *SEN1* is also involved in other pathways, including oxidative stress and SA-mediated plant defense (Oh et al., 1996; Schenk et al., 2005). Taken together, this may explain why *Arabidopsis* plants in the absence of microbes showed signs of abiotic stress and senescence, especially in the cotyledons (Figure S1).

### Photosynthesis

Microarray data analysis of *Arabidopsis* shoots and roots showed that genes required for photosynthesis (e.g., *RBCS* and *CAB*) were up-regulated in plants grown with soil microbes (Table S2). In addition, GO terms related to photosynthesis were enriched in roots and shoots, including response to light stimulus (GO:0009416), C fixation (GO:0015977), and photosynthesis (GO:0015979) (Tables 1, S3). This finding is consistent with the study by Zhang et al. (2008) who showed that the PGPR *B. subtilis* GB03 augments the photosynthetic capacity of *Arabidopsis* plants by decreasing glucose sensing and ABA levels. Growth promotion and increased photosynthesis have also been reported for *Phaseolus vulgaris* and rice when inoculated with phosphorus solubilizing bacteria and *Sinorhizobium meliloti* 1021, respectively (Collavino et al., 2010). A higher photosynthetic efficiency was also conferred by endophytic bacteria to sugar beet (Shi et al., 2010). Common to the above studies is that they report an increase in growth and photosynthetic capacity of plants inoculated with individual microbial strains. The present study indicates that increased leaf growth and photosynthesis could still be observed when plants were exposed to whole soil microbial communities, possibly a result of the synergistic activities of a number of PGPR.

It is well documented that increased photosynthesis can also lead to higher oxidative stress in leaves (Hideg and Schreiber, 2007) and an increase of the GO term “Respiratory burst” could be observed in microarray data from plant leaves grown in the presence of microbes (Table 1). This includes some genes that encode proteins associated with oxidative stress in leaves, for example NADPH oxidase (*RBOHD*), MAPK3, and peroxidases 21 and 71 (Table S2; Figure 5B). Interestingly, this is in contrast to genes encoding other peroxidases (peroxidase 42, 50), catalase 1, ERF6, ZAT10 and SEN1 that were down-regulated in leaves in the presence of microbes (Figure 5B; Table S2). While there is no simple explanation for this observation, this adds to the growing body of evidence showing that redox homeostasis and ROS production are associated with many different processes in the plant where different gene family members also play different roles and are often involved in many other functions (Miller et al., 2008).

### Plant defense and beneficial interactions

The GO term enrichment analysis revealed the major biological processes involved in roots and shoots when exposed to compost soil-derived whole microbial communities (Tables 1, S3). JA and ET signaling were upregulated in the presence of microbes especially in shoots, which was evidenced by the genes involved in these processes that contributed to the enrichment of the GO terms “response to biotic stimulus” (e.g., *CYP71A12, CYP83B1, ERF104*) and “response to stress” (e.g., *WRKY38, ERF2, WRKY54, ATL2, JAZ6, At1G32920*). The fact that such responses occurred mainly in shoots indicates that they are systemic rather than local, given that most of the interactions in this study were more likely to be occurring underground (Van Wees et al., 2008; Van der Ent et al., 2009). Although jasmonate and ethylene signaling have been associated to ISR and the recognition of specific strains of beneficial microbes (Matilla et al., 2009; Alizadeh et al., 2013; Chowdappa et al., 2013), it appears that these signaling pathways also play a major role in recognizing microbes at the community level. This suggests that these interactions are more frequent than previously thought. Certain microbe-derived molecules are recognized by plants as non-self through receptors and elicit the MAMPs-triggered immunity (MTI, Zamioudis and Pieterse, 2012). MAMPs include flagellin, lipopolysaccharides (LPS), peptidoglycans, and elongation factor Tu (Pel and Pieterse, 2012). MTI responses such as production of ROS are elicited by non-symbiotic microbes at first (Zamioudis and Pieterse, 2012). As the observed outcome of the system that we were investigating represents a combination of a multitude of interactions with different microbes, the induction of several genes involved in oxidative stress in shoots may be a net result of these initial encounters that continuously occur during the plant's lifecycle with microbes in the environment. However, these microbes are also believed to actively suppress this initial defense response by utilizing effector molecules and hormone-like compounds (Zamioudis and Pieterse, 2012). Effectors include low molecular weight molecules and LPS. Another strategy used by some bacteria to avoid recognition by the plant host is to reversibly switch between phenotypic stages (Pel and Pieterse, 2012; Zamioudis and Pieterse, 2012). This process is called phase variation (Van der Woude, 2011). Thereafter, usually just a mild systemic immune response is elicited and this prepares the host for future pathogen attacks which is then combated more promptly and intensively, also referred to as priming and that is also effective against insect attacks (Conrath et al., 2006; Pieterse and Dicke, 2007). Interestingly, genes involved in indole glucosinolate biosynthesis, which is believed to play a role in plant responses against insect attack (Agerbirk et al., 2008), were upregulated in shoots (*MYB51*, *CYP83B1*, Table 1). JA signaling has also been associated with a reduction in ROS (Ton et al., 2002; Pieterse et al., 2009), mainly for two scenarios: (1) to prevent harm towards beneficial microbes and (2) to prevent cell death when attacked by a necrotrophic pathogen. JA signaling is also generally regarded to be antagonistic to SA and ABA signaling, two pathways that also involve ROS production for protection against biotrophic pathogens and abiotic stress, respectively (Anderson et al., 2004; Kazan and Manners, 2013). A recent 16 S rRNA pyrotag sequencing study on *Arabidopsis*-acclimated rhizosphere soil suggests that plants under normal conditions attract growth-promoting bacteria, while during conditions of JA-mediated plant defense, soil bacteria with antimicrobial and insecticidal attributes were enriched (Carvalhais et al., 2013). Further experimentation should focus on the effect of different types of whole soil microbial communities on plant growth promotion and ISR, a promising area of research that may lead to increased crop yields and effective biocontrol of pathogens and pests.

### Multifunctionality during multiple interactions

Many genes that were up or downregulated in the presence of microbes have multiple functions. This was apparent in this study in particular for genes involved in signaling, including those that encode transcription factors (e.g., *MYB72, WRKY25*) and genes required for redox homeostasis (e.g., the peroxidase-encoding gene family and *ERF6*). Gene expression profiling, including the genome-wide microarray data (Table S2) can serve as a platform to provide additional leads on functionality. However, ultimately, the genetic approach (use of mutants, up or downregulation of genes or gene families) in combination with physiological analyses should be used to determine plant function during multiple plant-microbe interactions. GO term analysis may provide an overview of some of the processes occurring in plants as it takes into account multiple known roles of genes (Table 1 and S3). For example, another functional GO term found to be overrepresented in the list of genes which were induced in roots in the presence of microbes was “Plant cell wall organization or biogenesis” (Table S3). *E. coli* and *Saccharomyces cerevisiae* are able to be taken up by root cells and serve as nutrient sources to *Arabidopsis* and tomato plants (Paungfoo-Lonhienne et al., 2010b). This process is accompanied by extensive modifications in root cell wall, including cell wall outgrowth, and enhanced expression of genes involved in cell wall modification (Paungfoo-Lonhienne et al., 2010b). Here, plants were exposed to a wider range of microbes, therefore it is possible that *Arabidopsis* roots have sensed microbes and regulated cell wall modification genes to uptake microbial cells into the roots and use them as a source of nutrients. However, mechanisms involved in this process and whether there is a preferential uptake of certain microbes as opposed to others still needs to be further investigated. Finally, it should be mentioned that, although *Arabidopsis* plants used in this study were checked for the presence of culturable microorganisms, the possibility cannot be excluded that plants may still have contained endophytic organisms (Bulgarelli et al., 2012).

To our knowledge this is the first study to use microbe-free soil to compare some of the main processes involved in interactions between plants and whole microbial communities. Iron acquisition, JA signaling, photosynthesis, redox homeostasis, and plant cell wall organization appear to be the driving mechanisms affected by *Arabidopsis* and rhizosphere microbial communities interactions. Although most previous studies have focused on individual plant-microbe interactions, multi-species analyses such as simultaneous plant and microbial metatranscriptomics coupled to metagenomics (Berendsen et al., 2012; Carvalhais et al., 2012; Delmont et al., 2012; Schenk et al., 2012) may be required to further increase our understanding of the intricate networks underlying plant-microbe interactions in their diverse environments.

### Conflict of interest statement

The authors declare that the research was conducted in the absence of any commercial or financial relationships that could be construed as a potential conflict of interest.

## Supplementary material

Table S1 **List of genes and primers used for qRT-PCR analyses**.

Table S2 **Genome-wide microarray data analysis of *Arabidopsis* plants grown in the absence or presence of whole soil microbial communities**. The complete data sets are shown for root and shoot tissues, as well as lists of differentially expressed genes (up and downregulated in the presence of microbes) that passed all selection criteria for data analysis (see Materials and Methods for details).

Table S3 **Identification of Gene Ontology (GO) functional categories that are enriched in transcript populations in roots in the presence of whole soil microbial communities**.

Figure S1 **Sampling of plant shoots, roots and rhizosphere soil**. First, plants were carefully uprooted, soil attached to roots (rhizosphere soil) was shaken off and sieved. Plants were then carefully washed to remove excess soil and briefly blotted onto tissue paper. Shoots and roots were sampled separately. All samples were immediately snap-frozen in liquid N_2_ before storage at −80°C. The photograph shows a typical plant grown in the absence of microbes. The red arrow indicates senescence at the cotyledons.

Figure S2 **Carbon-nitrogen (C/N) ratios in whole plant tissues from *Arabidopsis* plants cultivated in the presence or absence of microbes**.

Figure S3 **ICP-OES analysis of total element concentration in sieved bulk soil from *Arabidopsis* plants grown in the presence or absence of soil microbes. (A)** Macronutrients, **(B)** Micronutrients. Bars represent mean values in mg/kg soil dry weight (DW) ±SD from 3 independent replicates (100 g soil pooled from 10 vessels/replicate).

Figure S4 **Comparison of *IRT1* and *MYB72* expression in *Arabidopsis* plants grown in the presence or absence of soil microorganisms using either autoclaved or γ-irradiated soil as the growth substrate**. qRT-PCR results from 3 biological replicates and SDs are shown.
