# Supplementary material for: Plant growth in Arabidopsis is assisted by compost soil-derived microbial communities
Source: Front Plant Sci. 2013 Jul 4;4:235. doi: 10.3389/fpls.2013.00235 (PMC3701873; doi:10.3389/fpls.2013.00235)
Supplement: Table S1 — List of genes and primers used for qRT-PCR analyses. [file DataSheet1.DOC]

**Supplementary Table 1.** List of genes and primers used for qRT-PCR analyses

| **Gene name** | **Gene locus** | **Primer Sequence (5' to 3')** |
| --- | --- | --- |
| *ERF6* | AT4G17490 | R - CATGCTCAGAAACTCCGTCAAATC  F – ACGGTGGTTGAGAAAGTGCTAAAG |
| *ERF104* | AT5G61600 | R - TCCCCTGTAGTGCCTCTCTTCTTC F - TAACCAGCGTAAACCGCCCTTACC |
| *MYB72* | AT1G56160 | R - CTGCTTTTGTGCTTTGGTCATG F - CAACGAGATCAAAAACGTGTGG |
| *WRKY70* | AT3G56400 | R - ACACGTCTCCGATCTCTTTTTTCT F - GTTTGAAGATTCCGGCGATAGTC |
| *LECTIN1* | AT3G15356 | R - GACCAAACTTTTCTTTTTCCGACTAA F - ATGGAAAGTCAGAAAACAACCTCATATT |
| *LECTIN2* | AT3G16530 | R - AATTCGATAGCCAAGATGTGGTTC F - GCCTTCATCATAACCCCGGAA |
| *PER50* | AT4G37520 | R - TTTATTGACATCGTCGGTCGG  F - CGTCGTTAATCTTGCGGGTG |
| *NIA1 / NR1* | AT1G77760 | R - ATCTCTGCGTGACCAGGTGTT F - CAAATTCCGAAGCTTGGTGG |
| *SEN1* | AT4G35770 | R - GTCGTTGCTTTCCTCCATCG  F – AACATGTGGATCTTTCAAGTGCC |
| *IRT1* | AT4G19690 | R - AAGAGCCGCGATTAAACACTTG F - GCTCATTTACATGGCATCGTG |
| *OPR2* | AT1G76690 | R - CATCAGTTCTGTCATTCACTTTGTCC F - ATGCTATGGAAGCTGGTTTTGATG |
| *PR1* | AT2G14610 | R - TGTAATTCCCCGGAGGAT  F - GTGGCGTGACTCGGTTCG |
| *ZAT10* | AT1G27730 | R - CCATCGAGAATTCAGGGATCG  F - GTGTCCAACTCCGAAGGTGC |
| *PDF1.2* | AT5G44420 | R - CGCAAACCCCTGACCATG  F - TTTGCTGCTTTCGACGCAC |
| *At3g07720* | AT3G07720 | R - CTCGTGATCCAGCTGCAAAC F – ACGCTGGAATGGAGGAAATG |
| *FRD1 / FRO2* | AT1G01580 | R - GCAAATGGTCAACTAAGCTTCACC F - TGCCTTAAGAAATCGGCGG |
| *OPT3* | AT4G16370 | R - TAGAGTAAGCATCACCACCACCG F - GAGTTTTAGCCTCAATCCTGGTCC |
| *MYC2* | AT1G32640 | R - AGCAACGTTTACAAGCTTTGATTG F - TCATACGACGGTTGCCAGAA |
| *MYB15* | AT3G23250 | R - CCAGCCACTTCTAGGTCATTCG F - CCTGATTGTGTTTCCAAGAAGATTG |
| *WRKY25* | AT2G30250 | R- CCTGATTGTGTTTCCAAGAAGATTG F - CAGGCTTAGGATGATTATGACCACC |
| *CAT1* | AT1G20630 | R - CGAGTTGCTAGTTTCTGTCCCAG F - CGTGAAGCGTTTTGTTGAAGC |
