# Supplementary material for: Plant growth in Arabidopsis is assisted by compost soil-derived microbial communities
Source: Front Plant Sci. 2013 Jul 4;4:235. doi: 10.3389/fpls.2013.00235 (PMC3701873; doi:10.3389/fpls.2013.00235)
Supplement: Table S3 — Identification of Gene Ontology (GO) functional categories that are enriched in transcript populations in roots in the presence of whole soil microbial communities. [file DataSheet3.DOC]

**Supplementary Table 3.** Identification of Gene Ontology (GO) functional categories that are enriched in transcript populations in roots in the presence of whole soil microbial communities.

| **GO Term** | ***P*-value** | **Sample frequency** | **Background Frequency** | **Gene Names** |
| --- | --- | --- | --- | --- |
| ***Upregulated*** | | | | |
| GO:0050896 response to stimulus | 3.90E-03 | 17/52 (32.7%) | 3485/29974 (11.6%) | SAG21, AT1G20620, AT4G13790, UBQ10, TIP2, AT3G32980.1, AT2G38390.1, ATEXT4, AT5G38430, MLP328, AT4G08770.1, AT5G38420, ATGSTF2, FSD1, RBCS1A, AT2G18150.1, CAB2 |
| GO:0009628 response to abiotic stimulus | 1.46E-02 | 9/52 (17.3%) | 1240/29974 (4.1%) | SAG21, AT1G20620, AT4G13790, AT2G38390.1, AT5G38430, AT5G38420, ATGSTF2, RBCS1A, CAB2 |
| GO:0006979 response to oxidative stress | 1.53E-03 | 6/52 (11.5%) | 251/29974 (0.8%) | SAG21, AT1G20620, AT3G32980.1, AT4G08770.1, FSD1, AT2G18150.1 |
| GO:0009416 response to light stimulus | 7.21E-03 | 6/52 (11.5%) | 495/29974 (1.7%) | AT1G20620, AT4G13790, AT5G38430, AT5G38420, RBCS1A, CAB2 |
| GO:0015977 carbon fixation | 2.57E-04 | 3/52 (5.8%) | 11/29974 (0.0%) | AT5G38430, AT5G38420, RBCS1A |
| GO:0071669 plant-type cell wall organization or biogenesis | 1.29E-03 | 5/52 (9.6%) | 137/29974 (0.5%) | AT2G24980.1, ATEXT3, AT5G06630.1, AT2G43150.1, AT1G26250.1 |
| ***Downregulated*** | | | | |
| GO:0006826 iron ion transport | 6.64E-04 | 4/163 (2.5%) | 14/29974 (0.0%) | IRT2, FRO2, AT3G61010.1, IRT1 |
| GO:0055072 iron ion homeostasis | 6.64E-04 | 4/163 (2.5%) | 14/29974 (0.0%) | FRD3, FRO2, AT3G61010.1, IRT1 |
