# Supplementary figures and images for: Plant growth in Arabidopsis is assisted by compost soil-derived microbial communities
Source: Front Plant Sci. 2013 Jul 4;4:235. doi: 10.3389/fpls.2013.00235 (PMC3701873; doi:10.3389/fpls.2013.00235)

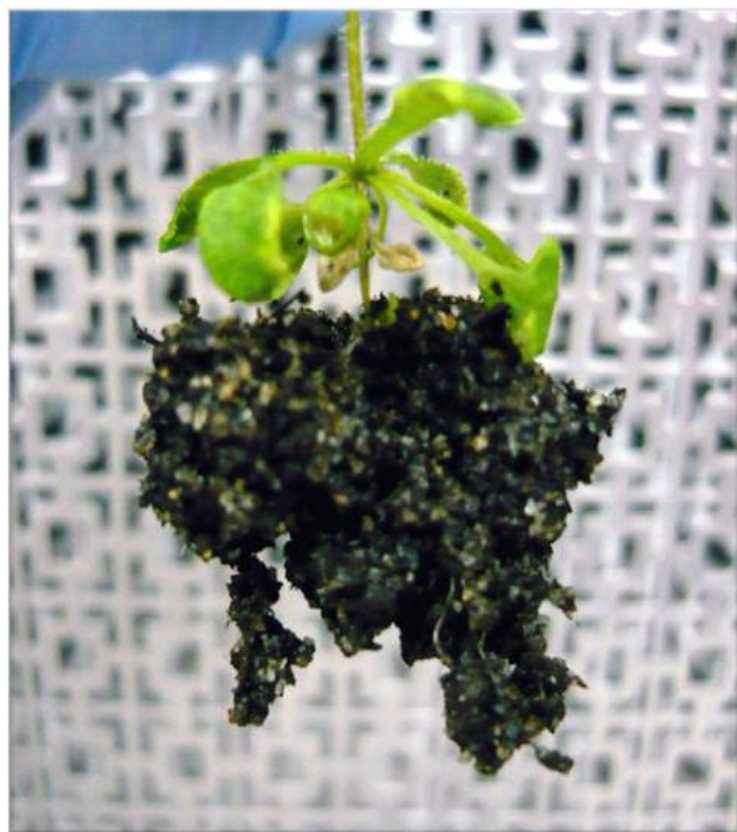

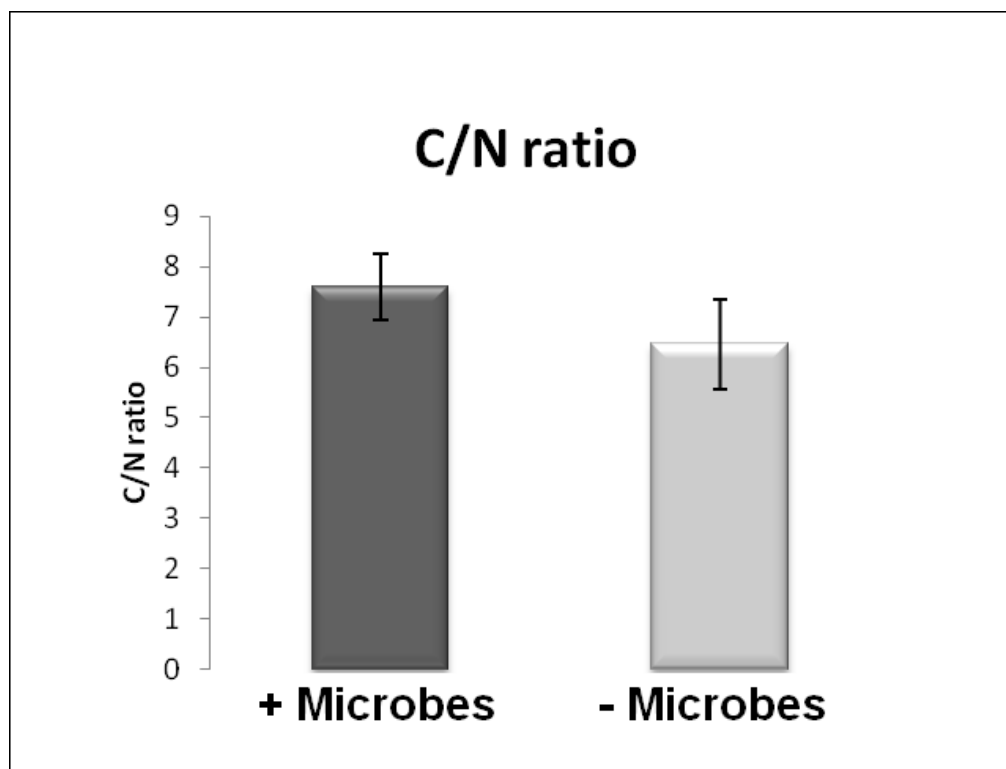

### Macroelements in bulk soil

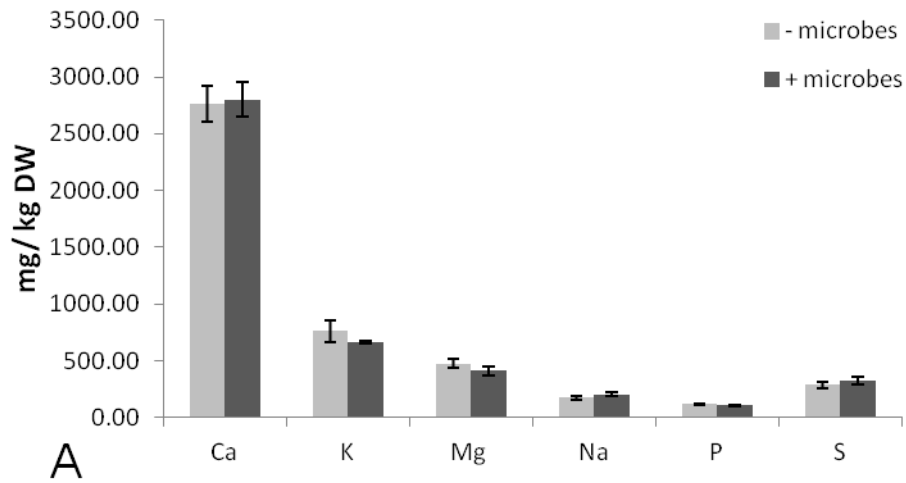

### Microelements in bulk soil

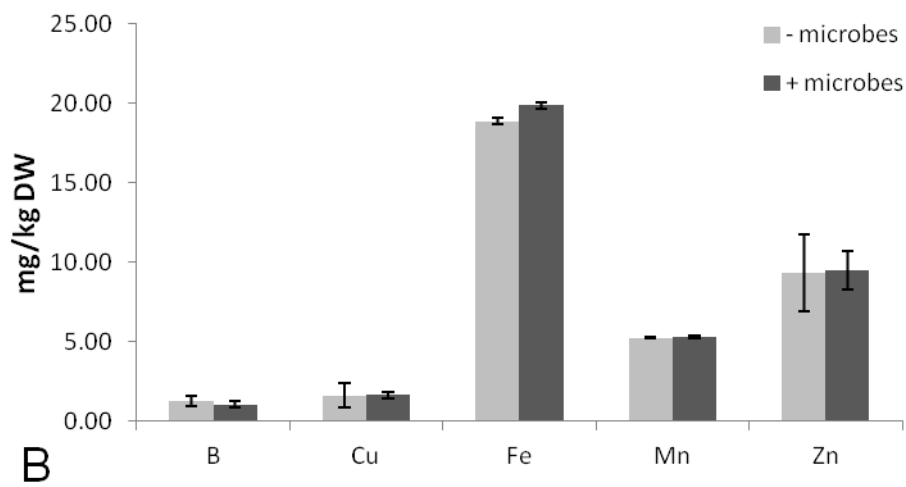

### *IRT1*

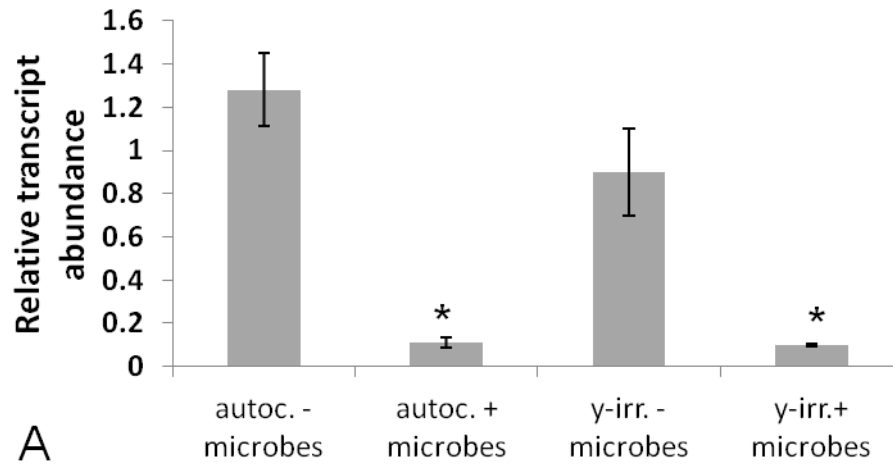

### *MYB72*

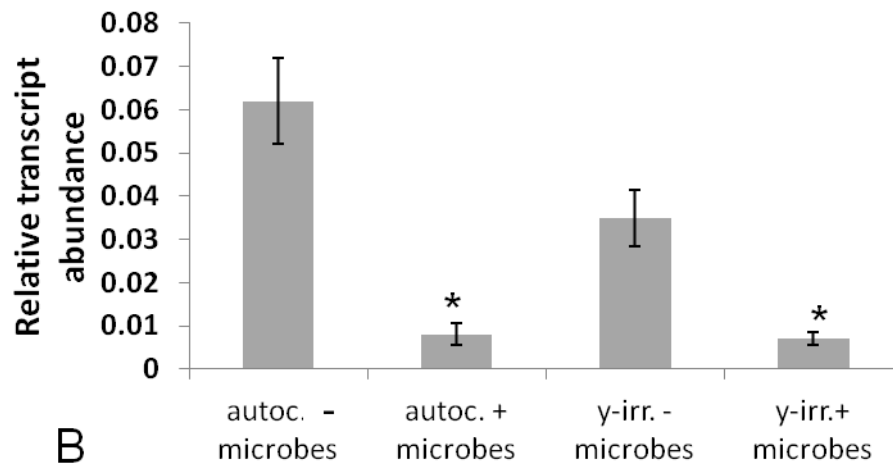

Supplement: Figure S1 — Sampling of plant shoots, roots and rhizosphere soil. First, plants were carefully uprooted, soil attached to roots (rhizosphere soil) was shaken off and sieved. Plants were then carefully washed to remove excess soil and briefly blotted onto tissue paper. Shoots and roots were sampled separately. All samples were immediately snap-frozen in liquid N2 before storage at −80°C. The photograph shows a typical plant grown in the absence of microbes. The red arrow indicates senescence at the cotyledons. [file Presentation1.PDF]
